# Imported leishmaniasis in Sweden 1993–2016

**DOI:** 10.1017/S0950268818001309

**Published:** 2018-05-31

**Authors:** S. K. Söbirk, M. Inghammar, M. Collin, L. Davidsson

**Affiliations:** 1 Division of Infection Medicine, Department of Clinical Sciences, Lund University, Lund, Sweden; 2 Unit for Parasitology, The Public Health Agency of Sweden, Solna, Stockholm, Sweden

**Keywords:** Epidemiology, leishmaniasis, parasitic infections, Sweden, travel

## Abstract

In Sweden, leishmaniasis is an imported disease and its epidemiology and incidence were not known until now. We conducted a retrospective, nationwide, epidemiological study from 1993 to 2016. Probable cases were patients with leishmaniasis diagnoses reported to the Swedish Patient registry, collecting data on admitted patients in Swedish healthcare since 1993 and out-patient visits since 2001. Confirmed cases were those with a laboratory test positive for leishmaniasis during 1993–2016. 299 probable cases and 182 confirmed cases were identified. Annual incidence ranged from 0.023 to 0.35 per 100 000 with a rapid increase in the last 4 years. Of 182 laboratory-verified cases, 96 were diagnosed from 2013 to 2016, and in this group, almost half of the patients were children under 18 years. Patients presented in different healthcare settings in all regions of Sweden. Cutaneous leishmaniasis was the most common clinical manifestation and the majority of infections were acquired in Asia including the Middle East, specifically Syria and Afghanistan. *Leishmania tropica* was responsible for the majority of cases (42%). A combination of laboratory methods increased the sensitivity of diagnosis among confirmed cases. In 2016, one-tenth of the Swedish population were born in *Leishmania*-endemic countries and many Swedes travel to these countries for work or vacation. Swedish residents who have spent time in *Leishmania*-endemic areas, could be at risk of developing disease some time during their lives. Increased awareness and knowledge are needed for correct diagnosis and management of leishmaniasis in Sweden.

## Introduction

Human leishmaniasis is caused by protozoan trypanosomatids from one of over 20 *Leishmania* species. The disease, transmitted by female phlebotomine sand flies, is endemic in over 97 countries in Asia, Africa, Southern Europe, South and Central America [1, 2]. Clinical manifestations of infection are determined by properties of the parasite (species and possibly strain) and by the immunological status of the host. Disease is classified as cutaneous (CL), mucocutaneous (MCL) or visceral leishmaniasis (VL), also known as kala-azar. Post-kala-azar dermal leishmaniasis (PKDL) can sometimes present after treatment of VL. Asymptomatic carriership of *Leishmania* parasites or latent infection may later lead to manifest disease in individuals who become immunosuppressed (e.g. due to transplantation or immunomodulatory therapy) and may also have implications for transfusion safety [3–5].

Leishmaniasis is a rare, imported disease in Sweden. Healthcare providers may easily miss cases if they are unfamiliar with the symptoms, signs and risks of acute or latent infection. In addition, patients may present with a variety of symptoms, often months after visiting *Leishmania*-endemic regions and they may seek healthcare in many different clinics throughout Sweden. Delayed diagnosis or incorrect treatment may lead to increased morbidity and mortality [6].

The incidence of imported leishmaniasis has increased in non-endemic countries in Europe in recent years due to increased travel to, and immigration from, endemic regions [7–9]. Leishmaniasis is not notifiable in Sweden according to the communicable disease act, and therefore, the incidence of imported leishmaniasis in Sweden is not known. However, Swedish healthcare providers are encouraged by the Public Health Agency of Sweden (PHAS) to be aware of possible leishmaniasis in travellers and immigrants from endemic countries [10].

The aim of the present study is to estimate the incidence of imported leishmaniasis in Sweden and to describe the clinical presentation, patient characteristics, a country where the infection was acquired and causative species.

## Methods

### Study design

We conducted a retrospective nationwide study in Sweden, from 1993 to 2016, linking individual patient data from prospectively compiled national healthcare registers with information in databases of diagnostic laboratories. We estimated the annual incidence of leishmaniasis and assessed clinical presentation, patient characteristics, probable country of infection and species distribution.

Ethical approval for all parts of the study was obtained by the regional Ethics Committee (Dnr 2014/646 + Dnr 2016/1101). All data were analysed anonymously.

### Data sources and data collection

We identified all patients diagnosed with leishmaniasis according to the International Classification of Disease (ICD) (Ninth version (ICD9): 085 or Tenth version (ICD10): B55), as recorded in the Swedish Patient Registry. This registry has two separate parts, the inpatient registry with data concerning patients registered during a hospital admission from 1993 to 2013 (PARSV = Patient Registry, Inpatients) and the outpatient registry where patients with a visit to a hospital clinic or an emergency ward were registered, from 2001 to 2013 (PAROV = Patient Registry, Outpatients). All visits with a diagnosis in the file since 2001 are supposed to be reported and the Patient Registry collects information about the patient's diagnosis, age, sex and date of visit, as well as location and type of reporting clinic [11].

*Leishmania* cases were also identified in the databases held by the Unit for Parasitology and Waterborne Pathogens at the PHAS from 1993 to 2016. These databases include information about the age, date of birth, sample type, date of sample collection, results of laboratory analyses, referring clinic, medical history and country where the infection was probably acquired. An attempt was made to identify cases from databases of 57 pathology and microbiology departments in Sweden. However, most of these laboratories did not analyse samples for leishmaniasis and only one of the departments, Clinical Microbiology Department in Skåne (southern part of Sweden), had searchable stored data about diagnostic samples for leishmaniasis, thus adding two extra confirmed cases. The cases from the databases at PHAS and Clinical Microbiology Department in Skåne constitute the laboratory-confirmed cases during 1993–2016. There was enough clinical data in the database of Public Health Agency to support the case definitions suggested by the WHO in all cases of confirmed VL and MCL [12]. All cases reported as CL have had positive confirmatory parasitological tests from skin smears or skin biopsies.

The Total Population Registry (Statistics Sweden) collects information about the age, sex, county of residence and country of origin for the residents of Sweden. (f.d. 10, Statistics Sweden) Information from this registry was used to calculate incidence rates and for retrieving information on the country of origin of Swedish residents. Since all Swedish residents are assigned unique personal identification numbers, information from the various databases was linked using these identifiers [13].

RESURS (http://www.tdb.se) has collected data about travel from Sweden up to the end of 2014. Travel data are collected by 24 000 telephone interviews each year, which record trips by Swedish residents between the ages of 2 months and 74 years old. From these data, we extracted trips with at least one overnight stay in 2014 made to *Leishmania*-endemic countries.

### Diagnostic analyses and species typing

Clinical samples for the diagnosis of leishmaniasis included serum, aspirates, biopsies and curettage from infected tissue. The diagnostic methods used were microscopy, *in vitro* culture, molecular detection and serology. Microscopy was in some cases performed at the local laboratory or department; however, microscopy results reported in this study were performed at The PHAS. A buffy coat was prepared from peripheral blood and bone marrow submitted in EDTA. Skin smears were fixed with 100% methanol prior to staining. Slides were stained with Giemsa and examined for amastigotes under 50× and 100× objectives. *In vitro* culture was performed at two sites: The PHAS or the Clinical Microbiology Department in Skåne. Samples submitted in RPMI-1640 transport medium or Novy–MCNeal–Nicolle medium (NNN-medium) were cultured for promastigotes at 23 °C for 4 weeks. The RPMI-1640 complete culture medium contained L-glutamine, HEPES (4-(2-hydroxyethyl)piperazine-1-ethanesulfonic acid, N-(2-hydroxyethyl)piperazine-N′-(2-ethanesulfonic acid), penicillin, streptomycin and fetal calf serum. All molecular and serological analyses were performed by the Unit for Parasitology and Waterborne Pathogens at The PHAS. An in-house immunofluorescence microscopy test using cultured promastigotes of *L. donovani* (MHOM/ET/67/HU3) was used to detect anti-*Leishmania* antibodies (IgA, IgM and IgG) in patient serum. Patient samples were diluted 1:10, 1:30 and so forth. The cut-off was 1:10. Over the years, species-typing methods have changed, as have their ability to discriminate between closely related *Leishmania* species. Prior to 2010, species typing was performed by testing cultured parasites with a panel of monoclonal antibodies from WHO (personal communication with Jadwiga Winiecka-Krusnell). From 2010 to 2016, this was replaced by two molecular methods: detection of *Leishmania* species DNA [14] followed by typing of the species using PCR and restriction fragment length polymorphism (RFLP analysis) [15].

The taxonomy of *Leishmania* species has been debated and modified over the years, especially with the advent of molecular methods. This study uses the taxonomical system for species, complexes, subgenera and genus as described by Van der Auwera and Dujardin, which is a modified version of the one previously described by Schönian *et al.* [16, 17]. The species identification method used by the PHAS from 2010 to 2016 (ITS1 PCR–RFLP [18]) has been compared with typing results from other European laboratories and is shown to be an accurate method, although discrimination of some species can only be made to the level of species complex (*L. donovani* complex) or subgenus [*L.*(*Viannia*)] [19].

We therefore report results at the species level for *L. major*, *L. tropica*, *L. aethiopica*, *L. mexicana* and *L. amazonensis*. *L. donovani* and *L. infantum* (also known as *L. chagasi*) are reported as *L. donovani* complex. The ITS1 PCR–RFLP method [18] cannot differentiate between species in the subgenus *Viannia*; so these are all reported as only subgenus *L. (Viannia)*.

### Data analysis

We analysed the distribution of demographic data and clinical characteristics and these data are presented as proportions or medians (interquartile range) where appropriate. Patients diagnosed with leishmaniasis before their 18th birthday were categorised as children. Data regarding the country where the infection was probably acquired were grouped into five regions: Africa, Asia, Europe, Central- and South America, where Middle Eastern countries were reported as Asia. We assessed the sensitivity for the various diagnostic methods to diagnose leishmaniasis (microscopy, culture, molecular and serology). Sensitivity was calculated as a number of patients positive for each analysis/number of analyses per number of patients for which the same analysis/analyses were performed. Any positive test is used as the golden standard.

A confirmed case was defined as a case identified in the databases of the diagnostic laboratories, which was confirmed by microscopy, culture, molecular methods or serology. A probable case was defined as a case identified in the National Patient Registry without any match in the databases of the laboratories, i.e. without microbiological confirmation.

The annual incidence of imported leishmaniasis was calculated from the total number of new cases with the annual Swedish population size as the denominator. Confidence intervals were calculated using the Poisson distribution. We used linear regression to estimate differences in annual incidence over the study period. Data were stored and analysed in Microsoft Excel 2010 and STATA/SE version 13.1. The distribution of cases in Sweden was illustrated with a map created with the online tool ArcGIS (https://www.arcgis.com/features/index.html).

## Results

### Incidence of imported leishmaniasis

In total, 299 cases of leishmaniasis were identified from the National Patient Registry from 1993 to 2013. This is probably an underestimate of cases, as the outpatient records only start from 2001 and data for both inpatients and outpatients are incomplete after 2013. Of these 299 cases, 93 could be identified among the verified laboratory-confirmed cases using the patients’ unique personal identification numbers. The remaining 206 patients were not found in the laboratory registers, so these constitute the probable (unconfirmed) cases, where samples were not submitted to the PHAS or Clinical Microbiology laboratory in Region Skåne. Out of 896 recorded visits due to leishmaniasis in the patient registry, 66 could not be linked to a complete personal identification number, so they have been excluded from the analysis.

In total, 182 laboratory-confirmed cases of leishmaniasis were identified from the laboratory databases used by the PHAS and the Clinical Microbiology laboratory in Region Skåne, from 1993 to 2016. Ninety-three of these cases were from 1993 to 2013 and 89 were from 2014 to 2016.

**Fig. 1.**
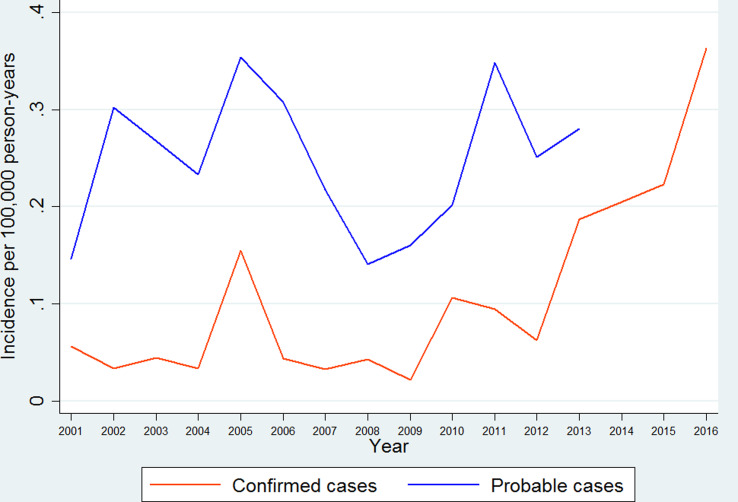
Annual incidence rates for probable and confirmed cases of imported leishmaniasis in Sweden 1993–2016.

We calculated the annual incidence of imported leishmaniasis separately for probable and confirmed cases. The annual incidence of probable cases ranged from 0.14 [95% confidence interval (CI) 0.14–0.14] per 100 000 person-years in 2008 to a maximum of 0.35 (95% CI 0.35–0.35) in 2005, as shown in Figure 1. The incidence showed an increasing trend as estimated by linear regression (*P* < 0.001). Data concerning probable cases for the period 2014–2016 were incomplete and have not been included. The annual incidence of confirmed cases ranged from 0.02 (95% CI 0.02–0.02) per 100 000 person-years in 2009 to a maximum of 0.36 (95% CI 0.36–0.36) in 2016, as shown in Figure 1. The incidence of confirmed cases increased through the study period as estimated by linear regression, (*P* for trend < 0.001). Incidence rates of imported leishmaniasis for the years for which data from the Patient Registry are complete (2001–2013) can be seen in Figure 1.

### Confirmed cases of imported leishmaniasis

#### Clinical presentation

Of the 182 laboratory-confirmed cases, 169 cases (92.9%) presented with CL, six cases (3.3%) with MCL, five cases (2.7%) with VL and one case with PKDL. One case, which was culture-positive, had missing data for clinical presentation and sample type. Most of the samples analysed were skin biopsies followed by curettage or aspirate from the wound.

#### Demographic data

Leishmaniasis occurred in both sexes and all age groups (range 1–80 years). Of the 182 confirmed cases, 104 (57%) were male and 75 were female. The number of children (<18 years) diagnosed with leishmaniasis increased dramatically during the last 4 years of the study period. From 2013 to 2016, 46% of the confirmed cases were children. The number of confirmed cases, female to male ratio and median age per 4-year period is shown in Table 1.

**Table 1.** Laboratory-confirmed cases of leishmaniasis in Sweden 1993–2016 (*n* = 182)

Years	1993–1996	1997–2000	2001–2004	2005–2008	2009–2012	2013–2016
Cases *n* = 182	8	8	16	26	28	96
Female: Male ratio	1:1.7	1:1.7	1:3	1:1.6	1:1.3	1:1.3
Children *N* (%)	1 (12.5)	1 (12.5)	3 (18.8)	3 (11.5)	6 (21.4)	44 (45.8)
Median age, years (range)	36.8 (3–80)	52.4 (6–78)	32.2 (10–63)	32.6 (2–64)	30.1 (1–62)	19.2 (1–71)

#### Country of infection and species-typing

**Fig. 2.**
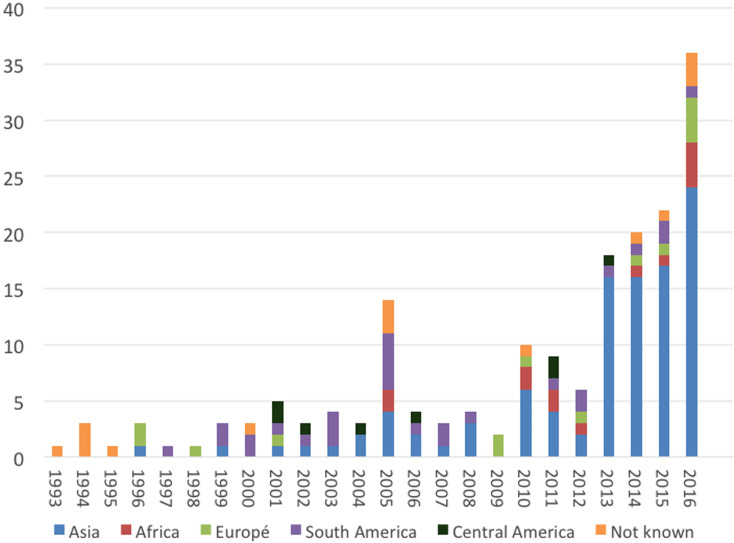
Imported leishmaniasis in Sweden, confirmed cases per year, region of infection 1993–2016.

The area where patients probably acquired the infection included 32 different countries. Information regarding the reason for stay in an endemic region was often missing in the databases and is therefore not reported. The countries of infection varied over the study period. There was a peak in confirmed cases in 2005 showing an increase in cases from South America and Asia. In 2010 the number of confirmed cases tripled compared to the year before. This increase in cases is probably due to the introduction of molecular diagnostic methods at the PHAS, which are used as a complement to microscopy, culture and serology. Then in 2013, the number of patients acquiring leishmaniasis in Asia increased markedly, as shown in Figure 2. Almost a third of confirmed cases (52/182) had Syria (The Syrian Arab Republic) as the probable country of infection and all of these were infected with *L. tropica* and presented with CL (Table 2). Afghanistan was the second most common country of infection with 35 cases; half of the cases were infected with *L. tropica* and the other half with *L. major*.

**Table 2.** Area where leishmaniasis was acquired and the infecting *Leishmania* species, 1993–2016

Probable region of infection The two countries with most cases within the region	*L .major*	*L .tropica*	*L .aethiopica*	*L. mexicana*	*L. donovani* complex	*L.(Viannia)* subgenus	Not typed	Total
**Africa**	**8**	**1**	**3**	**0**	**1**	**0**	**0**	**13**
Tunisia	5	0	0	0	0	0	0	5
Ethiopia	0	0	3	0	0	0	0	3
**Asia** (incl. Middle East)	**22**	**71**	**0**	**0**	**3**	**0**	**6**	**102**
Syria	0	50	0	0	0	0	2	52
Afghanistan	16	14	0	0	1	0	4	35
**Europe**	**0**	**1**	**0**	**0**	**12**	**0**	**0**	**13**
Spain	0	0	0	0	5	0	0	5
Greece	0	0	0	0	3	0	0	3
**South America**	**0**	**0**	**0**	**0**	**0**	**27**	**0**	**27**
Ecuador	0	0	0	0	0	8	0	8
Peru	0	0	0	0	0	6	0	6
**Central America**	**0**	**0**	**0**	**2**	**1**	**7**	**0**	**10**
Costa Rica	0	0	0	0	1	4	0	5
Panama	0	0	0	0	0	2	0	2
**Not known/missing**	**1**	**4**	**0**	**0**	**5**	**0**	**7**	**17**
Total	**31**	**77**	**3**	**2**	**22**	**34**	**13**	**182**

The most common species of *Leishmania* infecting patients was *L. tropica* (77 cases), followed by *L. (Viannia)* subgenus (34 cases) and *L. major* (31 cases). Over 50% (12/22) of the *L. donovani* complex infections were acquired in either Spain or Greece, so the likely infecting species is *L. infantum* [7, 20].

Among our confirmed cases were Swedish patients with TNF*α* modulating therapy for rheumatic disease infected with leishmaniasis while visiting a treatment clinic in Spain.

#### Diagnosing clinic

**Fig. 3.**
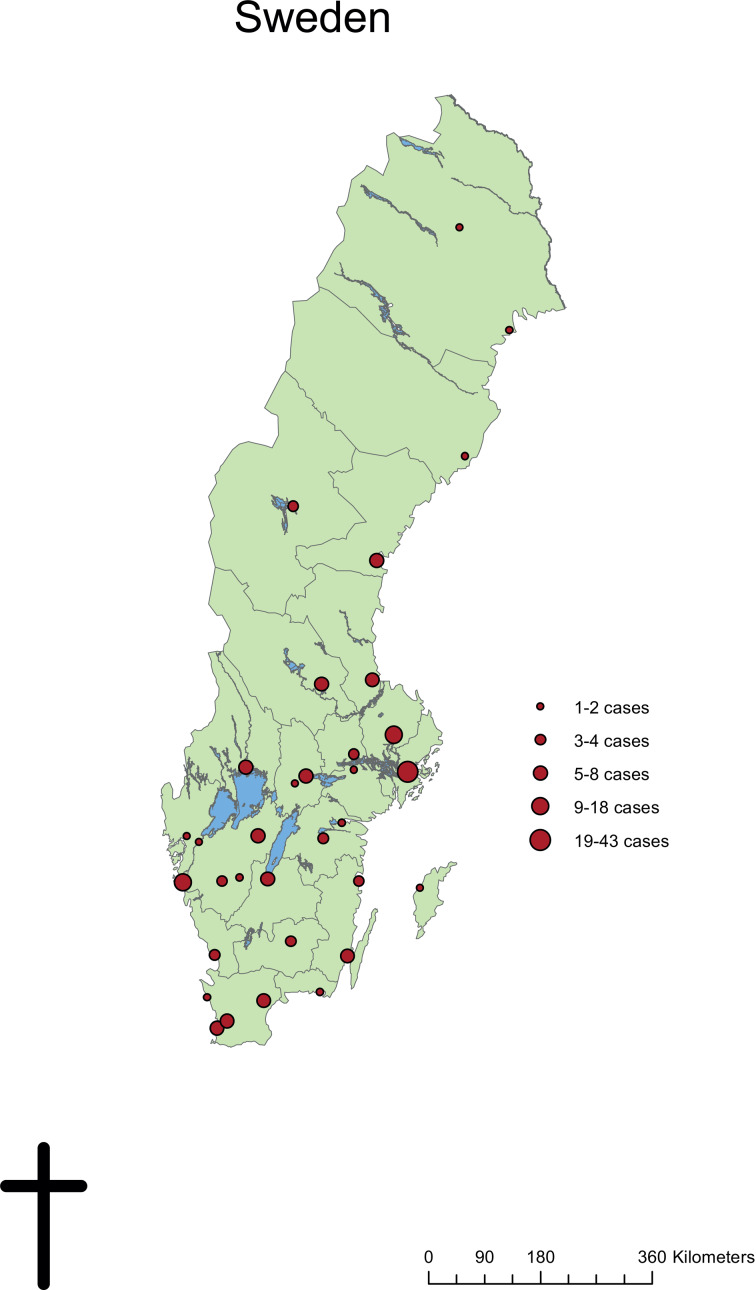
Geographical distribution of confirmed *Leishmania* cases from diagnosing clinics in Sweden, 1993–2016.

The most common specialty to submit patient samples for laboratory diagnosis were Departments of Infectious Diseases (100 patients, 54.9%), followed by Dermatology departments (70 patients, 38.5%). Other specialties with more than one laboratory-confirmed case were Paediatric departments (seven patients, 3.8%) and Primary healthcare units (four patients, 2.2%). Patients presented with leishmaniasis in 33 cities spread throughout Sweden. The geographical distribution of cases was not concentrated at the University Hospitals but corresponded to the population size of each region and city (Fig. 3).

#### Diagnostic methods

The sensitivity of different methods to diagnose leishmaniasis was compared using data from the PHAS. Sensitivity for each method within the group of confirmed cases is calculated as the number of patients positive for each analysis per number of patients for which the same analysis/analyses were performed. Any positive test was used as the golden standard. PCR was the single most sensitive method for the diagnosis of leishmaniasis, as 98% (122/124) of samples tested by PCR were positive. Sensitivity of the PCR method may have been improved by the fact that many of the samples had been transported in culture-medium, thus giving the parasites time and conditions to multiply before extraction of DNA. The sensitivity of microscopy (76%) was similar to that published in studies from the Netherlands (73%), Israel (74%) and the UK (62–77%) [7, 21, 22]. A combination of methods, as recommended by the WHO [12] (microscopy, culture and/or PCR and serology) increased sensitivity within the group of confirmed cases to 100%, as shown in Table 3.

**Table 3.** Sensitivity of PCR, microscopy and culture for diagnosis of leishmaniasis

Diagnostic methods	Positive	Negative	ND^a^	Sensitivity (%)^b^
Molecular: PCR	122	2	58	98
Microscopy	85	27	70	76
Culture	112	8	62	93
Microscopy and/or culture^a^	78	3	101	96
Microscopy and/or PCR^a^	80	1	101	99
Culture and/or PCR^a^	67	0	115	100
Microscopy, culture and/or PCR^a^	50	0	132	100

Data from the Public Health Agency of Sweden, 1993–2016 (*n* = 182 patients).

a Patients for whom not all methods were performed or results noted were included in the group labelled as ND (not done).

b Sensitivity is calculated as number of patients positive for each analysis or number of analyses per number of patients for which the same analysis/analyses were performed. Any positive test is used as a golden standard.

Antibody detection (serology), using immunofluorescence microscopy, was performed on serum from 50 patients, 37 of which were positive. One patient with suspected VL was positive with PCR from bone marrow only, whilst microscopy and culture from bone marrow were negative and there were no detectable antibodies. All other patients with VL, MCL or PKDL, who had serum samples tested, had detectable antibodies (Table 4). Most patients with CL were not tested for the presence of antibodies, but for the 41 who were, 29 (71%) were positive and most of their results were just over the limit for detection for the serological assay (cut-off 1:10). The CL patients with positive serology were infected with different *Leishmania* species including *L. donovani* complex (four cases), *L. (Viannia)* subgenus [14], *L. major* [2] and *L. tropica* [5].

**Table 4.** Epidemiology and diagnostic analyses for VL, PKDL and MCL cases

Non-CL cases *N* = 12	Infecting *Leishmania* species	Probable country of infection	Results of laboratory analyses			
			Molecular: PCR	Microscopy	Culture	Serology (serum titre^a^)
VL case no 1	ND^b^	ND	ND	Pos^c^ (LG)	Pos (LG)	Pos (270)
VL case no 2	*L. donovani* complex	Vietnam	Pos (BM)	Pos (BM)	Neg (BM)	Neg
VL case no 3	*L. donovani* complex	Italy or Spain	Pos (BM)	Pos (BM)	Pos (BM)	Pos (30)
VL case no 4	ND	Greece	ND	Pos (BM)	Pos (BM)	Pos (30)
VL case no 5	*L. donovani* complex	Spain	Pos (BM)	Pos (BM)	Pos (BM)	Pos (2430)
PKDL case	*L. donovani* complex	Montenegro (treated for VL)	ND	ND	Pos (TB)	Pos (270)
MCL case no 1	*L.V.* subgenus	Peru	ND	Pos (TB)	Pos (TB)	Pos (10)
MCL case no 2	*L.V.* subgenus	Ecuador	Pos (TB)	Neg	Pos (TB)	Pos (30)
MCL case no 3	*L.V.* subgenus	South America	ND	ND	Pos (LG)	Neg
MCL case no 4	*L. donovani* complex	Cameroon	Pos (TB) Neg (BM)	Neg	Neg (BM)	Pos (30)
MCL case no 5	*L. donovani* complex	Greece	Pos (TB)	Pos (TB)	ND	Pos (270)
MCL case no 6	*L. donovani* complex	Spain	Pos (TB)	Pos (TB)	ND	ND

a Serology cut-off 1:10.

b Patients for whom the analysis was not performed or specific data not available labelled as ND (not done).

c Pos, positive; Neg, Negative; LG, lymph gland aspirate or biopsy; BM, bone marrow aspirate or – biopsy; TB, tissue biopsy from mucosal, labial or skin tissue.

### Swedish population at risk for leishmaniasis

Swedish residents who have spent time in *Leishmania*-endemic areas could be at risk of developing the disease. In 2016, 1.8 million people living in Sweden (17.9% of 10 million) were born abroad, 952 000 of them (9.5% of the Swedish population) were born in *Leishmania*-endemic countries [1, 23]. Moreover, many Swedish residents travel to *Leishmania*-endemic countries for work or vacation purposes. According to travel data from Sweden collected by RESURS (http://www.tdb.se), 42% of the trips made by Swedish residents with at least one overnight stay in 2014 were made to *Leishmania*-endemic countries. Swedish residents made 16.3 million trips in 2014, and of the 6.8 million trips with *Leishmania*-endemic countries as final destination, only 0.5 million trips were work-related.

## Discussion

The purpose of our study was to describe and estimate the incidence of imported leishmaniasis in Sweden over a 20-year period. We present the most accurate estimate to date of the yearly incidence of imported leishmaniasis in Sweden. Although very low (under 0.5/100 000 person-years), the incidence of confirmed cases, which increased rapidly within the last years of the study period, had not yet peaked in 2016. Over the study period, from 1993 to 2016, 182 cases were laboratory-confirmed and almost 100 of these were diagnosed in the last four years. This represents more than a threefold increase in leishmaniasis cases. Half of these were in children under 18 years of age. CL was the most common clinical manifestation and the majority of infections were acquired in Asia, specifically Syria and Afghanistan. The species responsible for the majority of cases was *Leishmania tropica* (42%). The marked increase in a number of cases and the fact that most of them were infected in Syria reflect migration due to the current armed conflict and a large number of asylum-seekers coming to Sweden in recent years (https://www.migrationsverket.se/Om-Migrationsverket/Statistik.html). The countries of infection and infecting *Leishmania*-species differ to those described in previous studies of imported leishmaniasis in other European countries, as they describe the situation before the conflict in Syria [9, 22, 24–26].

Although all visits in hospital-based outpatient clinics should be reported to PAROV, the reports are not complete from all cities for every year, which can explain why we have some confirmed cases which we cannot identify in the PAROV and PARSV-registers. Of 896 registered clinic and hospital visits for leishmaniasis, 830 visits were linked to 299 patients with complete personal identification numbers. Recently arrived immigrants may not yet have received their personal identification number and could not be included in the statistics for probable cases. Leishmaniasis patients without a complete personal identification number constituted 66 (7%) of all recorded visits during the study period, 1993–2016.

Patients presented in different healthcare settings in all regions of Sweden. A patient with suspected leishmaniasis would not be managed by a primary healthcare centre in Sweden, but rather referred to a hospital-based outpatient clinic. However, some cases seen by the few private dermatologists in Sweden may have been missed, as well as all cases where leishmaniasis was never suspected. The national healthcare registers PARSV and PAROV, from which we have collected data on probable cases, do not include visits in the primary healthcare, and therefore some cases never seen in a specialist outpatient clinic, may have been missed in our data. However, as only 4 (2.2%) of the confirmed cases of Leishmaniasis had been diagnosed in the Primary Health Care, we believe that very few probable cases (where leishmaniasis has been suspected) are missed in our data. However, especially VL can easily be misdiagnosed. Our study will have missed all cases of VL, MCL and CL where suspicion was never raised and the patient did not receive the correct diagnosis and treatment. Prior to the implementation of molecular methods in 2010 in Sweden, not all samples were sent for species-typing. Cases only confirmed by microscopy at the local laboratory, may have been missed in our statistics of confirmed cases, but should, at least between 2001 and 2013, be included in the probable cases. This may explain why so few of the probable cases were identified among the confirmed cases. The sensitivity of microscopy in our study was only 76%, despite being performed by experienced staff at the parasitology laboratory of the PHAS. Our results indicate in accordance with previous studies that microscopy alone is not satisfactory to exclude leishmaniasis [7, 21–23]. A combination of laboratory methods increased the sensitivity in our material for diagnosis and also provides the possibility to identify the infecting species. *In vitro* culture is necessary when the number of parasites in the specimen is low or to detect viable parasites in cases of relapse. Molecular analyses offer information about the infecting *Leishmania* species, which can be used to guide the choice of treatment and patient follow-up. Although serology is not recommended for the diagnosis of CL, it had been performed in 41 of the 169 cases of CL, and the fact that 71% had weak positive results may reflect the large proportion of *L. tropica* and *L. (Viannia)* subgenus in the CL-group.

*Leishmania* parasites can survive for a long time after infecting an immunocompetent person and result in an asymptomatic infection. If the person's immune system becomes weakened, for example through TNF*α* modulating therapy, a serious infection can develop. In Sweden, the use of immunomodulatory drugs to treat several conditions is increasing. It is therefore important that we identify which patients have a higher risk of infection with *Leishmania* parasites to ensure that they receive appropriate screening before beginning treatment with immunomodulatory drugs, and the right diagnosis if they later present with symptoms of leishmaniasis. WHO recommends that practical laboratory tools should be developed to identify markers of infection and states that input is needed also in the field of diagnostics for asymptomatic carriers [23]. The patients with TNF*α* modulating therapy infected with leishmaniasis while visiting a treatment clinic in Spain enlightens the importance of increased suspicion of leishmaniasis and further studies in this group of patients.

In 2016, one-tenth of the Swedish population were born in *Leishmania*-endemic countries, and many Swedes travel to these countries for work or vacation purposes. Swedish residents, who have spent time in *Leishmania*-endemic areas, may be at risk of developing disease sometime during their lives. Many patients with leishmaniasis will probably present within the primary healthcare or paediatric healthcare, where diagnosis and correct management is dependent on knowledge about the disease. Increased awareness and knowledge is needed for the correct diagnosis and management of this rare, imported disease in Sweden.
